# A Treatise on Corns, Bunions, the Diseases of the Nails, and General Management of the Feet

**Published:** 1846-03

**Authors:** 


Art. VI.—*A Treatise on Corns, Bunions, the Diseases of the Nails, and General Management of the Feet.* By Lewis Durlacher, Surgeon Chiropodist (by special appointment) to the Queen. Philadelphia: Lea & Blanchard. 1845. (12mo. pp. 134.)

Mr. Lewis Durlacher, Surgeon Chiropodist to the Queen, feeling that the ailments of our nether extremities have been given over long enough to "ignorant and impudent empirics," has made an effort in this little volume to bring them within the pale of science. He thinks, with St. Paul, that the "head cannot say to the feet, I have no need of you," and that since they are necessary to the man, they are deserving of some portion of his care—worthy, at least, of being kept clean. We agree most heartily with the Chiropodist, and it shall not be our fault, if his earnest voice in behalf of science, and the feet, is raised in vain. We have faith in his candor; for he claims to have discovered no infallible cures. Thus he confesses that he has not found a panacea for corns, although he has devoted nearly thirty years to the search, "and has tried various chemical and other remedial agents." Very naturally he has his fears, "that the public will be somewhat disappointed at the paucity of the remedies" he proposes. The public is taken with polypharmacy, especially if the cures are all *specifics;* or else runs to the other extreme, and takes up with Homœopathy, or the *no* remedy system. And for the reason that he shuns both extremes, Mr. Durlacher stands many chances of failing with the public. But our purpose was not to indulge in such remarks as these, but to give some account of his book.

It opens with a preface, which is followed by an introduction, and in this the author goes into some learned disquisitions on the structure of the skin, referring those who are curious respecting the literature of corns, warts, &c., to the works of Hippocrates, Celsus, Scribonius Largus, Oribasius, Octavius, Horatianus, Aetius, Actuarius, Nonnus, Avicenna, Haley Abbas, Phases, and Alsaharavius. Our curiosity we confess is satisfied with Mr. Durlacher's treatise; others may dive into this sea of learning if they have a fancy for it.

Having finished his preface and introduction the body of the treatise follows, which is divided into thirteen chapters. These treat of the cause and growth of corns; hard corns; callosities; soft corns; festered corns; neuro-vascular corns; vascular excresence; bunions; nails and their diseases; chilblains; the management of the feet.

Of the manner in which the author treats these several topics, we cannot convey so just an idea by any other method, as we shall do by a few extracts. It will be seen that he gives dignity to his subject, and that he is entirely master of it. He has described the various kinds of corns, and comes to lay down the treatment for the several varieties, when he proceeds with the following description:

"The treatment for the common induration is exceedingly simple. It requires only to be carefully removed by scraping, or cut off with a small instrument made like a scalpel, and afterwards covered with any mild gum or soap plaster.

"Those corns which are formed on the projecting points require to be more carefully attended to, and should be extracted by dissecting round them between the sac and the corn, following its form until the whole of it is detached, avoiding injuring the sensitive skin beneath.

"In cases where the corn is of a conical shape, while dissecting it out, the instrument should be inclined so much inwards that the point shall closely follow the diminishing circumference of the corn to its apex.

"The corn which is situated at the outer edge of the nail, and is imbedded in the semilunar pellicle, demands great dexterity in its removal, as it is very thin and deeply seated, without any surrounding thickening.

"In operating on corns growing under the great toe-nail, the first thing to be done is the removal of as much of the nail as is loose, which can be effected with very little pain, with an ordinary nail-knife, pair of scissors, or nail-nippers, by first slitting it up on each side with the nippers or scissors, and removing the portion of nail thus partially separated with the knife, so as to expose the corn, which is then to be extracted in the usual way.

This form of corn, being produced by accidental causes, when properly extracted seldom or never returns.

"In those cases, where there is a corn situated in the inner flap of the great toe, it is necessary to remove several layers of the cuticle previously moistening them, until the corn is exposed, when it should be extracted. If it extends beneath the nail, a part thereof must be cut away, until the corn is distinctly seen, and then it should be taken out.

"It would be difficult to give more explicit instructions for the performance of these operations. Dexerity in the use of the instrument, in these as in every other operation connected with surgery, can only be obtained by practice and experience. This remark applies generally to the extraction of all kinds of corns."

This work, while it is professional, is not meant to be confined to the profession; nor ought it to be. The management of the feet—of the feet of children, especially—must be left very much to families; and great suffering and inconvenience in after life would be prevented, by the dissemination of the knowledge contained in this little volume among parents. However one may be disposed to smile on reading the title-page, we venture to say that no one who goes through the work will lay it down without a feeling of respect for the good sense, learning, and candor of its author.

